# Mental Health Professionals’ Views on the Influence of Media on Self-Harm in Young People: A Critical Discourse Analysis

**DOI:** 10.3390/healthcare13141640

**Published:** 2025-07-08

**Authors:** Tharushi Denipitiya, Annette Schlösser, Jo Bell

**Affiliations:** 1 Rotherham Doncaster and South Humber NHS Foundation Trust, South Yorkshire DN4 8QN, UK; 2 Clinical Psychology, University of Hull, Hull HU6 7RX, UK; a.schlosser@hull.ac.uk; 3 Psychology and Social Work, University of Hull, Hull HU6 7RX, UK; j.bell@hull.ac.uk

**Keywords:** healthcare professionals’ views, self-harm, suicide, media, young people, critical discourse analysis

## Abstract

**Background:** Self-harm in young people is influenced by multiple factors, with media playing a significant role. While research has examined its harmful and protective effects, little attention has been paid to how healthcare professionals interpret and respond to media’s role in shaping young people’s experiences of self-harm. To our knowledge, no research has examined adolescent mental health professionals’ perspectives and, crucially, how these are constructed and understood. The study aimed to examine the following: (1) how mental health practitioners construct and use discourses to interpret the role of media in young people’s self-harm; and (2) how these discourses shape clinical understanding and practice. **Methods:** This qualitative study employed semi-structured interviews with ten clinicians from child and adolescent mental health services across England working with young people who self-harm. Data were analysed using critical discourse analysis to uncover how broader societal and institutional narratives shape clinicians’ perspectives. **Results:** Two dominant discourses were identified: “Media as Disruptor” and “The Hidden World of Youth”. These discourses framed media as both a risk factor and a potential intervention tool, positioning media as a powerful yet morally ambiguous force in young people’s lives. Clinicians largely framed media’s influence as negative but acknowledged its capacity for education and intervention. **Conclusions:** This research offers new insights into how media-related self-harm risks and benefits are framed and managed in mental health care settings. The study underscores the need for systemic changes in clinical practice, enhanced training, updated guidelines and a shift towards broader sociocultural perspectives in understanding self-harm and suicidal behaviour.

## 1. Introduction

The National Institute of Care Excellence (NICE) [1] defines self-harm as an act of self-injury or poisoning that is intentional, regardless of the assumed intention or motivation, as an expression of emotional distress. Current NICE guidelines [1] also consider suicide attempts with little or no suicidal intent as self-harm when used to communicate distress or alleviate internal tension.

Despite the NICE guideline’s [1] definition of self-harm being widely used in clinical practice in the UK, the research literature highlights an overlap between self-harm and suicidal behaviours, and distinguishing between them brings challenges [2]. Self-harm can be defined as injuring or poisoning oneself, regardless of intent [3] or without suicidal intent [4]. Suicidal behaviour can be defined as a behaviour to end one’s life with suicidal intent [5]. For the purposes of this article, the terms self-harm and suicide will be used interchangeably to refer to deliberate self-injurious behaviour, regardless of suicidal intent.

Over the past two decades, self-harm rates among young people in the UK have risen significantly. In the early 2000s, the issue gained prominence with reports like Truth Hurts [6], reporting that 6.7% of young people aged 11–25 had self-harmed between 2004 and 2006. By 2014, public and professional awareness of self-harm had increased. National surveys showed rising rates of self-harm among young people, compared to earlier studies [7,8]. By 2018/19, 24% of 17-year-olds reported self-harming in the past year, with 7% stating they had self-harmed with suicidal intent during their lifetime [9]. Moreover, from 2012 to 2021, hospital admission rates for self-harm among 10–24-year-olds rose significantly from 400 per 100,000 to a peak of 550 per 100,000 [10]. These trends highlight the prevalence of self-harm and the urgent need for support for children and young people who self-harm (YPSH) in the UK.

Self-harm in young people is influenced by a complex interplay of psychological, social and environmental factors [9,11]. Research suggests that adverse childhood experiences (ACEs) can have a lasting impact, shaping individuals’ coping mechanisms and increasing their vulnerability to psychological distress well into adulthood [12]. In addition to early-life adversities, more immediate stressors—such as feelings of isolation, interpersonal conflicts, academic or occupational pressures and exposure to self-harm—have been identified as key triggers for self-injurious behaviours among young adults [13]. These findings highlight the multifaceted nature of self-harm, reflecting both deep-seated emotional struggles and the broader challenges of emerging adulthood.

Furthermore, self-harm serves a range of functions, from expressing distress to attempting emotional regulation or exerting a sense of control in moments of uncertainty [14,15,16]. These motivations underscore the profound interplay between internal turmoil and external stressors, reinforcing the need to consider both individual experiences and wider societal influences when examining self-harming behaviours.

### 1.1. The Role of Media in Self-Harm and Suicide: A Growing Concern

One of the most important wider societal influences is media, which has become increasingly embedded in young people’s everyday lives over recent years, shaping their perceptions, behaviours and interactions with the world. Research [17] highlights that media portrayals of self-harm and suicide—whether through news reports, fictionalised content or social media—may inadvertently amplify risks for vulnerable individuals. Studies suggest that the normalization and sensationalisation of self-harm in media contribute to heightened risk [18,19]. Research further emphasises the impact of highly publicised portrayals on suicide rates [20]. For example, the release of the television series 13 Reasons Why [21], which depicted the suicide of a young person, was associated with an increase in suicide rates among young audiences in the US.

The impact of media on suicidal behaviours has been commonly understood through two key foundational concepts: the Werther Effect [22] and the Papageno Effect [23]. The Werther Effect describes how exposure to suicidal behaviours in media can increase suicide rates by encouraging imitation; a phenomenon supported by research indicating that sensationalist portrayals can exacerbate suicidal behaviours [24,25]. Conversely, the Papageno Effect highlights the potential of responsible media reporting to prevent suicide by promoting narratives of resilience, coping strategies and recovery [23]. Together, these perspectives illustrate the dual role of media in both contributing to harmful behaviours or serving as a protective factor through constructive portrayals.

Beyond media portrayals, the nature of young people’s media use itself is a growing area of concern. Bell and Westoby [26] argue that today’s media landscape is far more pervasive and interactive than ever before, leading to increased exposure to self-harm-related discourse and content. This shift has heightened accessibility to unmoderated content, making the media’s role in influencing self-harm behaviours more powerful and difficult to manage [27]. A striking example of this is the case of Molly Russell, a 14-year-old girl in the UK who died by an act of self-harm after prolonged exposure to distressing online content. The 2022 inquest into her death concluded that she had suffered from “the negative effects of online content” [28], marking the first time social media influence was explicitly linked to a cause of death.

However, the media’s influence on self-harm is not wholly negative. Digital spaces also provide young people with avenues for support, education and community [29,30,31]. Online forums, mental health advocacy campaigns and personal recovery narratives have been found to offer valuable support for young people experiencing distress. While some forms of media content may glamorise or reinforce self-harm, others contribute to awareness, prevention and access to professional help.

Recent reviews of international research reflect this duality in the role of media influence on YPSH [32,33,34,35,36]. A systematized review [32] found that YPSH are more active on social media than their peers, often using these platforms for connection and support, while also being at risk of harmful exposure and psychological distress. Similarly, a narrative review [33] highlighted both risk and protective factors across 38 studies, noting social media’s role in exacerbating self-harm for vulnerable young people but also its potential for suicide prevention.

Despite the well-documented dual influences of media on self-harm among young people, there remains a pressing need for professionals working with this population to deepen their understanding of media’s impact and to integrate this knowledge into clinical practice [29,33,37]. Recent scholarship has shifted focus in this direction. For example, a large-scale meta-analysis [34] reinforced the importance of focusing on the *nature and quality* of online engagement. A practitioner-oriented systematic review [34] underscored the need for open, developmentally appropriate clinical conversations to mitigate harm while respecting young people’s autonomy. Consistent with these findings, a scoping review [36] called for a more nuanced, balanced and context-sensitive approach to research and practice.

While prior research has examined the influence of media content on YPSH, there is a marked absence of research exploring how healthcare professionals themselves make sense of this influence in clinical settings. Critically, clinicians’ perspectives not only shape the quality of care and support provided to young people [38,39], but also influence how risk is assessed, managed and responded to within services. Despite the centrality of clinician perspectives in shaping care pathways, no existing studies have specifically explored how adolescent mental health professionals conceptualise and respond to media’s role in self-harm, or how these understandings are discursively constructed within broader institutional and sociocultural contexts.

This study directly addresses this gap. To our knowledge, it is the first study to examine adolescent mental health professionals’ perspectives on the influence of media on YPSH through a social constructionist lens. In doing so, it offers a novel contribution to the field by shifting focus from media content and adolescent behaviours to the interpretive frameworks clinicians use. Given the increasing rates of YPSH [10], the rising number of YPSH engaging with mental health services [40] and the increasing awareness of media’s role as a risk factor, this research is particularly timely. Insights from this study could inform interventions, risk assessment frameworks and professional training to better address media-related risks.

### 1.2. Exploring Media and Self-Harm Through a Social Constructionist Lens

A social constructionist approach provides a valuable framework for examining how the influence of media on YPSH is understood and constructed in clinical discourse. Social constructionism posits that knowledge and meaning are shaped by social interactions, language and cultural contexts [41]. This perspective challenges fixed or essentialist understandings of self-harm, instead emphasising how discourse, power dynamics and historical context shape how self-harm is framed and responded to in clinical practice.

Four key aspects of social constructionism—language, cultural and historical specificity, discourse and power dynamics—are particularly relevant to this study. Language is central to how self-harm is understood, as it determines how experiences are represented and interpreted [42]. Cultural and historical specificity highlights how perceptions of self-harm evolve over time and differ across societies. Discursive and disciplinary power shape dominant narratives, influencing whether self-harm is framed as an individual pathology, a social phenomenon or a consequence of systemic issues [29]. Finally, power dynamics play a critical role in clinical practice, as professionals hold authority in defining risk, vulnerability and appropriate interventions [43].

This study contributes to the existing literature by offering new insights into the influence of media on YPSH through a social constructionist lens. It aims to examine the following: (1) how mental health practitioners construct and use discourses to interpret the role of media in YPSH; and (2) how these discourses shape clinical understanding and practice. By analysing the discourses used by mental health professionals, the study advances current knowledge in three key ways. First, it shifts analytical focus from the impact of media on young people to the ways in which mental health professionals construct meaning around media’s role in self-harm, a perspective that has been largely overlooked in the existing literature. Second, by applying a social constructionist lens, it challenges dominant, individualised understandings of self-harm [44] and foregrounds the sociocultural and institutional discourses that shape clinical practice. Third, the study provides practical insights into how these discourses influence clinical responses, potentially informing more reflective and context-sensitive approaches to care.

## 2. Materials and Methods

This is a qualitative study using semi-structured interviews. Participants were 10 English-speaking clinicians from child and adolescent mental health services (CAMHSs) across England (3 males; 7 females). All had at least one year’s experience of working with YPSH within a CAMHS setting. They were recruited through purposive and snowball sampling [45].

The study did not collect data on participants’ age, race/ethnicity, education level and sexual orientation as the focus was on discourse rather than individual characteristics [46]. As individual characteristics were not under examination, in the interest of participant anonymity, it was deemed best not to collect the information [47].

Interviews were conducted via Microsoft Teams and recorded with clinicians’ consent. Interviews lasted approximately one hour, and clinicians were debriefed afterwards. The interviews were designed to provide rich qualitative data, offering insights into how clinicians construct and interpret media’s influence on YPSH. Questions explored clinicians’ roles and experiences of working with YPSH, their workplace practices and their perspectives on how societal and media narratives shape attitudes towards self-harm. Key areas of discussion included the following: young people’s engagement with media (news, television, film, social media and the internet) and its perceived impact on self-harm; how media is discussed within clinical settings and the extent to which it is considered in assessments and interventions; clinicians’ perceptions of media portrayals of self-harm and how these representations influence public and professional attitudes; the influence of clinicians’ own perspectives on their approach to care; direct experiences with media-related self-harm cases and reflections on how media exposure may contribute to young people’s self-harming behaviours.

### 2.1. Ethics

The University of Hull’s Faculty of Health Sciences Research Ethics Committee and the Health Research Authority provided ethical approval for the study.

### 2.2. Data Analysis

Data from the interviews were analysed using critical discourse analysis (CDA) [48]. CDA aligns with the social constructionist approach of the study, allowing for examination of the data through the perspective that an individual’s reality is shaped through discourse with both internal reflections and external interactions [41]. Using CDA allowed the researchers to examine constructions of meaning surrounding the influence of media on self-harm [49].

Interviews were transcribed verbatim and analysed according to the seven-step CDA process [48]. The professional roles of the interviewees were noted. Transcripts were then coded, and overarching discourses were identified. Similarities across transcripts and any positionalities the interviewees suggested were noted. Internal relations in the transcripts were highlighted by analysing any patterns, words or linguistic devices depicting social context, interviewees’ positionalities and power relations [48]. Finally, major discourses and the internal and external relations were interpreted. During this process, the researcher noted their reflections on how their perspectives may have influenced the analysis, questions, gaps and any insights [48].

### 2.3. Reflexivity

This study is grounded in relativist ontology and social constructionist epistemology, viewing knowledge as shaped by context and social interaction [41,50]. These informed the use of qualitative methods and CDA. At the time, the lead researcher who undertook data collection was a trainee clinical psychologist. Their clinical experience with YPSH and CAMHS professionals informed the research focus and helped build rapport. Ongoing reflexivity was maintained through journaling and regular supervision to manage potential bias [51]. The co-authors (a clinical psychologist and an academic) supported the lead researcher through regular, reflexive supervision and contributed to data analysis, interpretation and writing through relativist and social constructionist lenses.

We acknowledge that our backgrounds—as clinical and academic professionals with varying degrees of proximity to the subject matter, from different generations and different cultural backgrounds, with different family/professional roles and responsibilities—inevitably shaped the research process. We consider our diverse perspectives a resource for generating rich, multi-layered interpretations, provided we remain vigilant to the risks of over-identification or blind spots. Our commitment to reflexivity, transparency and collaborative analysis was central to ensuring the rigor of our qualitative inquiry [52].

## 3. Results

Data analysis revealed two dominant discourses: “Media as a Disruptor” and “The Hidden World of Youth”. These discourses cover the impact of media on YPSH and reflect clinicians’ perceptions as well as highlighting how language (including metaphors and constructions) positions YPSH within the context of the influence of media and self-harm.

### 3.1. Media as a Disruptor

The discourse of media as a disruptor appeared across all interviews. This discourse emphasises how media is seen as a powerful system influencing young people’s behaviours and perceptions relating to self-harm. The analysis revealed several sub-discourses within the main discourse: “Ease and Facilitation of Access”, “Multifaceted Nature of Media”, “Inaccuracy of Portrayals” and “Pathologising Behaviours”.

#### 3.1.1. Ease of Facilitation of Access

In this sub-discourse, media is portrayed as a facilitator of dangerous knowledge, with all clinicians expressing concern over the unrestricted access young people have to harmful content relating to self-harm. For example,

*“….it allows young people access to information that they shouldn’t really have. It’s not monitored. You know, some parents don’t feel like they have the control to put boundaries in place …and they have access to information. They have forums where they can find out how to self-harm. They get tips, and chat rooms. So I think it has a real influence on young people.”*

(clinician 2, CAMHS crisis consultant, female)

The clinician used lexical choices such as “shouldn’t really have”, “not monitored” and “real influence” to indicate the danger of easy access to media. The repetition of the verb “access” highlights how easy it is to access harmful information relating to self-harm, whilst the phrases “find out how to self-harm” and “get tips” indicate a direct link between media and the facilitation of self-harm behaviours. The use of modal verbs like “should” and “can” implies a prescriptive stance on what is appropriate for young people, which positions the media as overstepping boundaries regarding easy access to inappropriate material relating to self-harm.

Clinician 5 further reinforces this perception:

*“So I feel like outside influences do impact their relationship with media and how often they access things that…might lead to them… self-harm in all might sort of start off that spiral that ends in self-harm.”*

(healthcare assistant in a CAMHS inpatient unit, female)

The phrase “outside influences” is a metonym for media, indicating external factors, such as friends, beyond parental or clinical control. The clinician’s use of the verb “spiral” metaphorically suggests a downward, uncontrollable trajectory that can be initiated by exposure to media. The hesitations and pauses (“…”) reflect the clinician’s struggle to articulate the complex process of media influence and highlights the deceptive nature of the exposure to media.

#### 3.1.2. Multifaceted Nature of Media

Clinicians also acknowledged the multifaceted role of media, framing its potential for both harm and benefit:

*“The the young person who was hiding or holding the medication….found out that if I take 1000 milligrams of so and so…. I’ll kill myself. I can die and that’s where…the information came from so I don’t know. Yeah, that’s quite alarming. At the same time, we’ve got young people on inpatient that belongs to a suicide pact… meeting total strangers…on social media and getting ideas about self-harming and…hurt themselves …but at the same time, there’s one or two young people who meet people online, and they’ve been advising or please talk to your doctor…ring your phone telling your mum about what is happening, so it’s kind of…it’s a two-way things….it’s it’s sometimes positive. And I’ve had examples of being positive at the same time. I’ve had examples of where it is quite negative and alarming.”*

(clinician 3, specialty doctor in a CAMHS inpatient unit, male)

The clinician above juxtaposed harmful and protective aspects of media. The repetition of “at the same time” aims to balance the multifaceted nature of media influence. The clinician’s narrative shifts from alarming scenarios (“hiding or holding the medication” and “suicide pact”) to more positive interactions (“advising” and “please talk to your doctor”). The lexical choice “alarming” contrasts with “positive”, implying the ambivalent impact of media.

This is echoed by clinician 6:

*“And I guess, you know, there’s lots of…access…to many different and…at many different kind of sites and apps and…I don’t even know how…how we keep track of them all, to be honest. So…I think…it I’m defensive about it…I think it can be really good when it’s used well and it can be the devil’s playground…a lot of the time. I then also think that there is an element of, actually, is there too much?”*

(core CAMHS practitioner, female)

The use of the metaphor “devil’s playground” describes the potential dangers of media, suggesting it is chaotic with a dubious reputation. The phrase “when it’s used well” acknowledges the positive potential of media, while the rhetorical question “Is there too much?” implies the overwhelming nature of the ever changing and growing landscape of media.

#### 3.1.3. Inaccuracy of Portrayals

This sub-discourse reveals how clinicians challenge dominant media representations of self-harm, exposing underlying ideological constructions that can misinform and stigmatise:

*“You know, it’s not, it’s not some some planned out you know like as you say romanticised event it’s it’s not anything like how it’s portrayed in that programme but for the young person you know they think that that’s what it’s like because that’s their only experience of being shown….whole thing, so definitely damaging.”*

(clinician 5, healthcare assistant in a CAMHS inpatient unit, female)

Clinician 5’s use of repeated negation (“not some planned out” and “not anything like”) functions as a discursive strategy to dismantle media narratives that romanticise self-harm. This repetition constructs a binary opposition between media portrayals and the clinician’s experiential knowledge, reinforcing the idea that popular representations are not only inaccurate but potentially harmful. The term “romanticised” operates as a loaded evaluative term, suggesting that media narratives may aestheticise or trivialise self-harm, transforming it into a consumable spectacle rather than a serious mental health issue. In doing so, the clinician articulated concern over media’s influence on young people, framing them as susceptible to these distorted narratives due to limited alternative representations (“that’s their only experience of being shown”).

The phrase “definitely damaging” further consolidates a discourse of harm, whereby the media is positioned as a powerful actor capable of shaping perceptions and, by implication, behaviours. This construction reinforces a broader ideological critique of media as a space where complex psychological realities are oversimplified and commodified.

*“Negative…Yeah, there’s still…Yeah, it’s interesting….there’s this real idea, or I guess the perception that somebody might have when … something like that is….put out there in the way that it is with a bias that that young person is damaged. Broken. There’s something you know, must be something, really….difficult that they’re going, you know. And I’m not saying there isn’t, but I guess the the kind of portrayal of that is always really, you know, really negative.”*

(clinician 7, clinical lead in a CAMHS crisis team, male)

Clinician 7’s fragmented syntax, marked by hesitations (“yeah…there’s this real idea, or I guess the perception…”) reflects the struggle in articulating an issue that is both ideologically charged and personally emotive. The clinician’s lexical choices—particularly “damaged” and “broken”—reveal the internalisation and critique of dominant discourses that frame YPSH through a pathologising lens. These metaphors signal a powerful ideological framing in which self-harm is equated with deficiency or malfunction, perpetuated through biased media narratives.

The repetition of the evaluative phrase “really negative” at the end of the extract intensifies the critique and highlights the pervasiveness of stigmatising portrayals. This functions not only as a critique of media bias but also as a call for a more nuanced and respectful representation of young people’s mental health in public discourse.

#### 3.1.4. Pathologising Behaviours

This sub-discourse also criticises media and how it can unnecessarily pathologise common behaviours in young people.

*“I feel….that there are more influencers getting involved with regards to mental health, which therefore means that mental health seems to be a very hot topic …and it makes people who may be struggling…but it’s a struggle that is in keeping with the situation, so it’s a normal emotional feeling, start questioning whether whether they’re suffering from mental health problems.”*

(clinician 2, CAMHS crisis consultant, female)

The use of the phrase “hot topic” suggests mental health difficulties are being sensationalised and unnecessarily pathologised in the media. The contrast between the phrases “mental health problems” and “normal emotional feeling” suggests that the media can blur the lines between young people’s typical behaviours and mental health difficulties which may lead to needless self-diagnosis.

This is also reinforced by clinician 9:

*“And I suppose, yeah, trying to sort of….get parents to be open to the idea that emotional instability in adolescence is really quite normal….but yeah, but because somebody had said it on the telly, that was, yeah, the kind of the battle that we had that month.”*

(CAMHS crisis assessment practitioner, female)

The term “emotional instability” is used as a nominalisation that abstracts and generalises the concept, while “quite normal” sets out to normalise young people’s behaviours. The clinician’s reference to “somebody had said it on the telly” implies the authoritative power of the voices of the media, which can override clinical advice and complicate therapeutic efforts.

### 3.2. Hidden World of Youth

This discourse focuses on how young people’s use and engagement with media (and associated difficulties) is often hidden from parents, carers and clinicians. It also reveals clinicians’ suggestions for strategies to address media influence and highlights the need for improvements in the approach to caring for YPSH. Three sub-discourses were developed: “Asking About Media in Assessments”, “Risk Assessments” and “Importance of Further Education”.

#### 3.2.1. Asking About Media in Assessments

All clinicians described the need to integrate discussions about media consumption into initial assessments with YPSH:

*“But yeah, I think it’s something that often it it will speak about it if they bring it up. I would say it wouldn’t be something that I would proactively discuss….No, but I feel like it probably should have to change. I don’t feel like… I probably should be better at that…..I think maybe just asking those questions more and just saying to people, because often it’s it’s offered by them. Really what what they want to share about their…media awareness and how much they pay attention to things…, so we don’t do assessments like that in our team typically. So that because of the nature of the way that we work…unless it was an identified problem, I wouldn’t naturally bring it up unless they did with me.”*

(clinician 8, mental health nurse in a CAMHS intensive community support team, female)

The clinician’s hesitation and self-reflection (“I would say it wouldn’t be something” and “I probably should be”) indicate an awareness of the gap in current practice and a recognition of the need for improvements and change to the care for YPSH. The phrase “proactively discuss” indicates that this approach is different from the previous reactive approach, suggesting a shift towards a more deliberate inclusion of questions related to media consumption in assessments with YPSH.

#### 3.2.2. Risk Assessments

This discourse on risk assessments reveals concerns about the length and rigidity, which can hinder rapport, therapeutic relationship building and engagement with YPSH as demonstrated by clinicians 1 and 4:

*“It is easy to have a questionnaire about risk… And you, you you can ask those questions… reading it from a clipboard or you can engage in a conversation… and know the know the bits that you need to hit to write a really good risk assessment, but without it feeling like the young person’s just being interrogated*

(clinician 1, CAMHS clinical lead, male)

Clinician 1 contrasted the impersonal nature of a “questionnaire” with the relational approach of a “conversation”. The repetition of “you” and the juxtaposition of “clipboard” versus “conversation” highlight the clinician’s preference for a more interactive and less intimidating method of assessment.

*“A risk assessment which at the moment in our services are very, very lengthy documents. So I think it’s almost gone back to that filling a full session on risk and I don’t think that’s necessarily been useful….I would say….yeah, I almost wish that in this setting it we…. we steer away from focusing solely on risk, but ensuring that the young person feels that their individual needs are still being met despite the risk, if that makes sense.”*

(clinician 4, CAMHS practitioner, female)

The clinician’s use of the phrase “very, very lengthy” and “filling a full session” implies how burdensome current risk assessments can be. The wish for a shift away from “focusing solely on risk” suggests a need for a more balanced approach that can address both the risk and the internal world of what matters for YPSH:

*“Yeah, and …. It can also, I guess, reinforce dynamics of infantilization and victimisation, and it can prevent clinicians from….seeing and sharing and celebrating the strengths that the child has and the you know the rest of their internal world, which might be rich and fulfilling and….you know, we can miss that and we can reinforce…the problem itself, inadvertently through through that sense of threat that we have and…yeah, I guess the proverbial kind of tail that wags the dog rather than the dog that wags the tail….kind of….that can be what happens, can’t it with these processes, that should be just the tip of the iceberg of what we’re doing and should be there to serve what we’re doing, but instead can become…the beginning and the end and the the middle of it all. Yeah, it’s a risk.”*

(clinician 10, clinical psychologist in a CAMHS children in care team, female)

The clinician elaborated on the negative dynamics that can arise from a rigid focus on risk. They used complex metaphors (“tail that wags the dog”) and idiomatic expressions (“tip of the iceberg”) to illustrate the unintended consequences of a risk-focused approach. The phrase “reinforce dynamics of infantilisation and victimisation” critiques how risk-focused assessments can diminish the power and strengths of young people. This in turn can affect the rapport building, therapeutic relationship and the care of the young person.

#### 3.2.3. Importance of Further Education

Clinicians highlighted the need for ongoing education and training to understand and explore the impact of media on YPSH:

*“I think it is definitely shown me how little I know about this area. And I think as you quite rightly said…having an understanding of what it is that you know of the world, the online worlds that young people are inhabiting…and the exposure, you know what it what they are exposed to is so crucial for actually being able to engage with them and for them to feel understood and…potentially disclose what’s going on so that it’s been really helpful on that on that front. And it’s made. It made me want to go and do my own research. Really. And, you know, I don’t know where that would begin because I’m sure it would lead to all of the things we’ve talked about that rabbit holes that. Yeah, but I think I think you’re right. Some training around this….sounds very appropriate, yeah”*

(clinician 10, clinical psychologist in a CAMHS children in care team, female)

Here, the clinician’s narrative constructs a position of professional inadequacy and uncertainty in the face of rapidly evolving media landscapes, as evidenced by the admission: “it is definitely shown me how little I know about this area.” This serves to highlight a perceived knowledge-power gap between clinicians and YPSH. The phrase “having an understanding…of the online worlds that young people are inhabiting” reflects the discursive separation of clinicians from youth digital cultures, implicitly constructing these online environments as distinct, unfamiliar and potentially threatening domains. The clinician drew a boundary between professional expertise and the lived experiences of YPSH, situating the latter as an area in need of increased institutional knowledge through “training”.

Repetition of the term “exposure” works to foreground media as an omnipresent and potentially harmful force, constructing a narrative in which young people are framed as passive recipients of media content, thereby requiring protection or interpretation by informed adults. This aligns with a paternalistic discourse that reinforces adult authority and positions youth as vulnerable.

The metaphor of “rabbit holes” is particularly revealing, invoking imagery of depth, confusion and danger. It contributes to a discourse of media as a chaotic and unknowable terrain, potentially undermining clinicians’ authority and simultaneously justifying the call for structured training and the discourse of professional uncertainty that legitimises further institutional investment in media-related education.

## 4. Discussion

Our findings align with and extend insights from the existing research that highlights the complex and ambivalent role of social media in YPSH [32,33,34]. These studies underscore the dual nature of media—a view echoed by clinicians in our study. Our work builds on this literature by offering a critical discursive lens, showing how UK mental health professionals construct media as simultaneously harmful and potentially supportive and how these constructions shape their clinical reasoning and interactions with YPSH. Our findings also resonate with practitioner-focused research [35,36], which has advocated for a more nuanced, context-sensitive understanding of media use and both risk and resilience. Importantly, our study brings added attention to how clinicians’ views may inadvertently underplay young people’s capacity for media literacy, resilience and self-regulation—factors that are increasingly recognised as protective in the literature. These parallels reinforce the relevance of our discursive approach in exploring how clinicians grapple with this complexity in practice.

Our analysis sheds light on two prominent discourses: “Media as a Disruptor” and “The Hidden World of Youth”, which encapsulate the complex relationship between media consumption and YPSH. These discourses reveal how language and underlying ideologies construct and shape the understanding of YPSH and their interactions with suicide- and self-harm-related media. The discourse of “Media as Disruptor” positions media as a powerful, almost omnipresent force that significantly influences young people’s perceptions and behaviours. By framing media as a “disruptor”, the language reflects a duality: media is both a source of risk (facilitating access to harmful content) and a tool for potential mitigation (educational and supportive content). This dual framing reflects broader societal anxieties about media’s influence and its moral ambiguity. The emphasis on media’s transformative influence constructs it as an agent of power, often overshadowing young people’s agency. This framing risks portraying them as passive consumers of media, subject to its influence, rather than active participants navigating and resisting harmful discourses. 

The sub-discourses of “Ease and Facilitation of Access” and “Inaccuracy of Portrayals” highlight how media shapes the normalisation and glamorisation of self-harm through content on media platforms, such as forums and chat rooms, where self-harm techniques are sometimes openly discussed and even encouraged. From a CDA perspective, these constructions reflect societal preoccupations with control and moral regulation. The language emphasises how repeated exposure to certain narratives or imagery desensitises viewers, making self-harm seem more acceptable or inevitable. This draws on a broader cultural discourse of media as a homogenising force, blurring the boundaries between deviant and normative behaviours [53]. However, these discourses often fail to fully account for young people’s awareness of these dynamics and their ability to disengage from harmful content.

The discourse of “Pathologising Behaviours” highlights how media frames self-harm in ways that reduce it to a clinical issue or spectacle. This could inadvertently marginalise the sociocultural and emotional complexities of self-harm, simplifying the phenomenon to fit media’s demand for sensationalised or digestible content.

Patalay and Fitzsimmons [9] and McManus et al. [11] identified the widespread use of media platforms as sources of both information and encouragement for self-harming behaviours among young people. These findings lend authority to the claims about the risks of media exposure to suicide-related content, while the inclusion of clinicians’ perspectives situates the discussion within a professional context. This interplay legitimises the argument while potentially marginalising alternative narratives, such as the empowering aspects of young people’s media use. While some media content sensationalises or glamorises self-harm, documentaries and educational content have the potential to raise awareness and connect individuals with support services [54,55]. This polarity highlights the importance of critically evaluating representations of self-harm in media while recognising that young people are not passive consumers but active participants who interpret, engage with and navigate media in complex ways. 

The discourse of “Hidden World of Youth” constructs a narrative of young people’s lives as inaccessible, veiled by the complexities of digital interactions: it highlights the often private, inaccessible or misunderstood aspects of YPSH lives, particularly in the context of their interactions with digital media. This “hidden world” encompasses online spaces, behaviours and social dynamics that YPSH engage in, which can be difficult for parents, carers and health practitioners to fully grasp due to the unique ways young people navigate and use social media. The framing of online spaces as “rabbit holes” or “devil’s playgrounds” employs metaphorical language that evokes danger, mystery and moral panic. By emphasising the “hidden” nature of young people’s worlds, the discourse implicitly critiques adult institutions—such as parents, carers and clinical systems—for failing to keep pace with youth culture [56]. This reveals a broader ideological tension between generations and their differing relationships with digital media [57]. Reframing this “hidden world” as a space of both vulnerability and empowerment opens possibilities for clinicians to better support young people’s resilience, recognising that not all online experiences are inherently pathological.

### 4.1. Clinical Implications

The findings highlight power dynamics, ideological underpinnings and systemic limitations inherent in current clinical approaches. The discourse of “media as a disruptor” reframes media not only as an external influence but as a critical factor shaping the lived experiences of YPSH. Media has significantly altered the environment in which young people are growing up, shaping their cognitive, social and emotional development in ways that are distinct from previous generations. Integrating discussions about media consumption into clinical assessments becomes a means of unearthing the “hidden world” of young people. This approach reflects the necessity of challenging reductionist clinical practices that prioritise overt risk indicators over nuanced understandings of complex social and emotional contexts. It also creates opportunities for clinicians to recognise and build upon young people’s media literacy and capacity for critical engagement, rather than assuming a position of vulnerability alone.

By exploring young people’s media interactions, clinicians can dismantle the taken-for-granted assumptions about media as a neutral or wholly negative force. Instead, media is positioned as a site of complex negotiation, where triggers, social identities and coping mechanisms intersect. This perspective invites a more collaborative approach that foregrounds young people’s agency, adaptability and reflexivity in their lives. Such inquiry disrupts conventional power dynamics in clinical settings, allowing young people to co-construct their narratives rather than having their experiences pathologised or oversimplified. 

The discourse surrounding risk assessments underscores the professional’s role in perpetuating institutionalised practices that prioritise efficiency over relational depth. These assessments often rely on procedural checklists, reflecting a broader institutional discourse of control and standardisation. The study challenges this framework, arguing for a shift towards risk assessments that foster dialogical engagement with YPSH. This shift calls for practices that privilege narrative depth and emotional resonance, disrupting the clinician−client hierarchy and enabling the co-production of meaning. Such practices also create space to explore how young people manage and interpret their own media environments, empowering them to reflect on their agency and strategies for navigating online risk. Clinicians should proactively inquire about young people’s media use, paying particular attention to the types of content they are exposed to, the frequency with which they engage and how it may influence their self-harm behaviours. Integration of these questions can help clinicians gain valuable insights for more targeted and holistic care plans. 

The absence of media-specific guidance in current NICE assessment and management guidelines reflects a gap in institutional discourses around self-harm and media. Importantly, although current NICE guidelines [1] provide an extensive framework for working with YPSH, guidance regarding the impact and role of media on self-harm is currently absent. While acknowledging the significance of social media and internet use amongst YPSH, the guidelines do not provide recommendations for interventions, safety planning, harm minimisation and clinician training regarding media use. By failing to explicitly address the role of suicide- and self-harm-related media, these guidelines inadvertently reinforce a discourse of omission, where media’s pervasive impact on young people is minimised or ignored. Updating NICE guidelines to include media-specific strategies for interventions and safety planning—such as personalised media safety plans, safe spaces and online resources for YPSH—would signify a discursive shift towards acknowledging the sociotechnical realities of self-harm. This shift would not only validate young people’s experiences but also challenge the medical and risk-oriented frameworks that currently dominate clinical practices. It would also recognise young people’s agency, whose insights and choices can inform more balanced, ethical and empowering responses to online content.

The discourse of “the hidden world of youth” emphasises systemic challenges within clinical practice and underscores the necessity of equipping clinicians with the skills to engage meaningfully with the evolving media landscape. Our findings advocate that NICE guidance should also include recommendations regarding training for clinicians–training which interrogates both the content and the context of media use, enabling clinicians to critically evaluate and respond to the cultural and ideological forces at play. Such training should also equip professionals to recognise and support young people’s strengths—such as their media literacy, peer support networks and self-help practices—as potential resources in care planning. This would support clinicians to engage in open conversations that recognise young people’s autonomy in navigating media and enable clinicians to work collaboratively with both caregivers and young people to develop strategies for safer media engagement. This cooperative approach promotes shared responsibility, ensuring that media use is managed in a way that prioritises well-being while respecting young people’s perspectives and experiences.

### 4.2. Limitations of the Study

Several limitations should be acknowledged, which may affect the transferability of the findings. The study relied on self-reported data obtained through a small number of semi-structured interviews, introducing the possibility of self-selection and social desirability biases [58]. Nine of the ten participants were recruited from a single region in England, resulting in a geographically limited sample; a larger and more diverse sample could yield a broader range of perspectives. The design of the study also precludes causal inferences regarding the relationship between media exposure and self-harm behaviours. Longitudinal quantitative research examining changes in media use and self-harm over time would offer a stronger basis for identifying causal pathways.

Finally, the study focused only on the perspectives of clinicians, neglecting the voices of YPSH and their parents or carers. Future research should seek to include these perspectives and investigate how YPSH respond to the clinician discourses identified here. Future research should also explore the efficacy of training programs tailored to address the challenges clinicians face in understanding “The Hidden World of Youth”, incorporating the perspectives of YPSH and evaluative research on training interventions. This would generate more specific recommendations to enhance clinical training for mental health professionals.

## 5. Conclusions

To our knowledge, this is the first study to employ a CDA approach to examine healthcare professionals’ perspectives on the influence of media on self-harming behaviours in young people and how these are constructed and understood. Through an analysis and interpretation of the discourses of “Media as a Disruptor” and “The Hidden World of Youth”, the findings highlighted how these discourses are constructed through language and reflect broader societal concerns, positioning media as a powerful yet morally ambiguous force in young people’s lives.

Clinicians predominantly framed media’s influence on YPSH as negative, emphasizing risks such as desensitisation, glamorisation, and the normalisation of self-harm. This framing, however, risks oversimplifying media’s role and neglecting young people’s agency in navigating their digital environments. The findings underscore the need for systemic changes in clinical practice, including the integration of media-specific enquiries into assessments, enhanced training to critically evaluate the influence of media and the development of updated guidelines that promote personalised media safety strategies.

## Data Availability

The data presented in this study are not available on request from the corresponding author due to ethical and confidentiality reasons.

## Abbreviations

The following abbreviations are used in this manuscript:

NICE	National Institute of Care Excellence
YPSH	young people who self-harm
ACE	adverse childhood experience
CAMHS	child and adolescent mental health services
CDA	critical discourse analysis
